# Molecular characterisation of osteoblasts from bone obtained from people of Polynesian and European ancestry undergoing joint replacement surgery

**DOI:** 10.1038/s41598-021-81731-5

**Published:** 2021-01-28

**Authors:** Dorit Naot, Jarome Bentley, Cluny Macpherson, Rocco P. Pitto, Usha Bava, Ally J. Choi, Brya G. Matthews, Karen E. Callon, Ryan Gao, Anne Horne, Gregory D. Gamble, Ian R. Reid, Jillian Cornish

**Affiliations:** 1 Department of Medicine, University of Auckland, Private Bag 92019, Auckland, 1142 New Zealand; 2 Middlemore Hospital, Counties Manukau District Health Board, Auckland, 1062 New Zealand; 3 Massey University, Auckland, 0745 New Zealand; 4 Department of Orthopaedic Surgery, South Auckland Clinical Campus, University of Auckland, Private Bag 92019, Auckland, 1142 New Zealand; 5 Department of Molecular Medicine and Pathology, University of Auckland, Private Bag 92019, Auckland, 1142 New Zealand; 6 Present Address: Australian Institute for Bioengineering and Nanotechnology, The University of Queensland, Brisbane, Australia

**Keywords:** Medical research, Molecular medicine

## Abstract

Population studies in Aotearoa New Zealand found higher bone mineral density and lower rate of hip fracture in people of Polynesian ancestry compared to Europeans. We hypothesised that differences in osteoblast proliferation and differentiation contribute to the differences in bone properties between the two groups. Osteoblasts were cultured from bone samples obtained from 30 people of Polynesian ancestry and 25 Europeans who had joint replacement surgeries for osteoarthritis. The fraction of cells in S-phase was determined by flow cytometry, and gene expression was analysed by microarray and real-time PCR. We found no differences in the fraction of osteoblasts in S-phase between the groups. Global gene expression analysis identified 79 differentially expressed genes (fold change > 2, FDR P < 0.1). Analysis of selected genes by real-time PCR found higher expression of *COL1A1* and *KRT34* in Polynesians, whereas *BGLAP*, *DKK1*, *NOV*, *CDH13*, *EFHD1* and *EFNB2* were higher in Europeans (P ≤ 0.01). Osteoblasts from European donors had higher levels of late differentiation markers and genes encoding proteins that inhibit the Wnt signalling pathway. This variability may contribute to the differences in bone properties between people of Polynesian and European ancestry that had been determined in previous studies.

## Introduction

Bone health is determined by a combination of factors, including age, genetics, body size and composition, lifestyle, and social determinants^1^. Ethnicity, a term relating to a person’s cultural affiliation, captures some of these factors, and clinical studies have demonstrated relations between ethnicity and bone health. The most common endpoints of these studies are areal bone mineral density (BMD) and fracture rate, although bone geometry and microarchitecture have also been investigated^1^.

The current knowledge of ethnic variations in bone properties and health are based on multi-ethnic studies, predominantly from the USA, and several smaller studies of different populations across the world. A large number of studies found that Black women and men have higher BMD and lower fracture rates than their White counterparts^2^. The largest multi-ethnic study to date is The National Osteoporosis Risk Assessment (NORA) study that determined peripheral BMD and new fractures during the following year in nearly 200,000 women of five different ethnicities^3^. The study found that Black women had the highest BMD and Asian women had the lowest, whereas White and Hispanic women had the highest risk for fracture. A common finding in studies that compared different ethnic groups is that higher BMD cannot fully account for reduced fracture rates. The NORA study found that after adjusting for multiple covariates, including weight, Asian women had similar BMD to White women, but their fracture risk was considerably lower. Another study found that the absolute incidence of fracture was lower by 30–40% in Black women in comparison to White women with similar BMD^4^. Bone properties in different ethnic groups have also been studied in Aotearoa New Zealand, a country with a large Polynesian population, comprised of Māori and Pacific peoples. Women of Polynesian ancestry have greater bone mineral content (BMC) and higher BMD than age-matched New Zealand European women, and femoral neck BMD was still about 15% higher in Polynesians after correction for bone size and body mass index^5,6^. Rates of hip fracture were also substantially lower in Polynesian people^7^.

Bone structure and material composition are determined by the activity of bone cells through the processes of modelling and remodelling. Three main cell types carry out these processes: osteoblasts that produce the mineralised bone matrix, osteoclasts that resorb bone matrix, and osteocytes that regulate modelling and remodelling of bone. Analyses of gene and protein expression in bone cells have been used to identify molecular mechanisms that underlie bone phenotype. A common source of tissue used in analysis of gene expression in human bone are samples obtained from patients undergoing joint replacement surgeries. As reviewed in Reppe et al.^8^, most studies compared gene expression between patients who had joint surgeries due to an osteoporotic fracture to those who had surgeries for osteoarthritis. Although differentially expressed genes have been identified, results vary between different studies, and further studies are currently underway^8^. Ethnic variations in the profile of gene expression in bone have not been investigated.

The current study focused on cellular and molecular mechanisms that could potentially contribute to the observed difference in bone properties between Polynesians and Europeans living in Aotearoa New Zealand. We hypothesised that differences in osteoblast proliferation and gene expression contribute to the differences in bone properties between the groups. We tested this hypothesis using bone samples obtained from patients undergoing total joint replacement surgeries for osteoarthritis.

## Results

### Study participants

Characteristics of the study participants are presented in Table 1. The mean age of the European group was significantly higher than that of the Polynesian group, whereas the mean body mass index (BMI) was similar in the two groups.

**Table 1:** Characteristics of the study participants.

	Polynesian	European	Difference between means^b^
n (F/M)	21/9	13/12	–
Age^a^ (years)	60.8 ± 11.8	68.76 ± 6.5	8.0 (2.7 to 13.3)*
BMI^a^ (kg/m^2^)	35.9 ± 6.3	33.0 ± 6.6	− 2.96 (− 6.5 to 0.6)

^a^Age and BMI data are presented as mean ± SD.

^b^Presented as difference (95% CI), *P = 0.004.

### Osteoblasts cultured from the two groups have similar S-phase fraction

**Figure 1:**
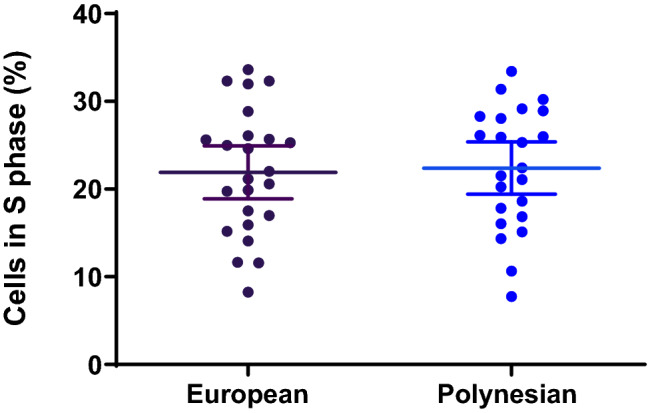
The percentage of cells in S‐phase in osteoblasts cultured from bone fragments. Cells were harvested at 50% confluence, labelled with propidium iodide and analysed by flow cytometry. The ModFit LT software was used to determine the fraction of cells in each phase of the cell cycle. The graph presents individual values, means, and 95% CI. Analysis by t-test found no significant differences between the groups. Figure created in GraphPad Prism 8.2.1, https://www.graphpad.com/.

In order to test the hypothesis that increased osteoblast proliferation contributes to the favourable bone properties of people of Polynesian ancestry that had been described in the literature^6,7^, the fraction of cells in different phases of the cell cycle were determined. Osteoblasts cultured from bone fragments were harvested at 50% confluence, labelled with propidium iodide and analysed by flow cytometry. The fractions of cells in S-phase were similar in the Polynesian and European groups (Fig. 1), indicating that when cultured, proliferation rates do not differ substantially between the groups. The fractions of cells in S-phase remained similar between the two groups in further analysis with covariate adjustment for age.

### Global analysis of differential gene expression by microarrays

Global gene expression in osteoblast cultures was determined by GeneChip PrimeView microarrays, using 10 RNA samples from each group. There was no significant age difference between these two groups of 10 participants. The signal distribution on the 20 microarrays and the principal component analysis are presented in supplementary Figures S1 and S2, and a scatter plot of the average level of gene expression from the two groups is presented in supplementary Figure S3. Of the total 79 genes that were differentially expressed (fold change > 2 and FDR-adjusted P value < 0.1), 51 genes had higher expression in the Polynesian group (Table 2) and 28 genes had higher expression in the European group (Table 3). Several of the genes that had the greatest difference in expression levels between the groups had been previously studied in the musculoskeletal system^9–11^. These include *HAS1*, a gene encoding hyaluronan synthase 1, which was 3.7-fold higher in the Polynesian group, and two genes that had higher expression in the European group: *MEGF10,* encoding the membrane receptor multiple EGF Like Domains 10, and *SEMA6D,* encoding semaphorin 6D, which were 5.2- and 3.2-fold higher, respectively.

**Table 2:** Genes with higher expression levels in Polynesians.

	Gene symbol	Fold change	FDR P value^a^		Gene symbol	Fold change	FDR P value
1	*FLG*	6.61	0.0697	27	*CLSTN1*	2.21	0.0067
2	*C1D*	5.3	0.0772	28	*DYNC1H1*	2.19	0.0079
3	*HAS1*	3.68	0.0473	29	*AKAP11*	2.18	0.0012
4	*MRPL43*	3.18	0.0076	30	*PLCB4*	2.18	0.0134
5	*PLPP4*	3.0	0.0537	31	*PSME4*	2.17	0.0158
6	*CCDC186*	2.9	0.0006	32	*DDX6*	2.16	0.0008
7	*DHX9*	2.73	0.0002	33	*IQGAP1*	2.15	0.0001
8	*PRRC2C*	2.72	0.0005	34	*UHMK1*	2.14	0.0136
9	*CDC27*	2.61	0.003	35	*HACD2*	2.13	0.0011
10	*KRT34*	2.61	0.0184	36	*PRDM2*	2.13	0.0005
11	*ZNF506*	2.61	0.0218	37	*CCND1*	2.12	0.0573
12	*ZFR*	2.59	0.0009	38	*CENPF*	2.11	0.0298
13	*COL4A2*	2.57	0.0185	39	*INHBA*	2.11	0.0346
14	*BAGE2*	2.55	0.0009	40	*NUFIP2*	2.11	0.0002
15	*MAP4K5*	2.54	0.0005	41	*LOC441155*	2.07	0.0016
16	*LAPTM5*	2.46	0.0002	42	*SETD2*	2.07	0.0024
17	*WAPL*	2.42	0.0001	43	*EIF4G1*	2.06	0.0037
18	*RBAK*	2.4	0.0001	44	*BOD1L1*	2.05	0.0016
19	*LPP*	2.38	0.0001	45	*SH3KBP1*	2.04	0.0019
20	*WNK1*	2.38	0.001	46	*ESCO1*	2.03	0.0009
21	*NFIX*	2.37	0.0002	47	*MAP4*	2.03	0.0007
22	*LYPD1*	2.35	0.0297	48	*MAP1A*	2.02	0.0007
23	*AKAP2*	2.29	0.0005	49	*AKT3*	2.01	0.003
24	*PRKDC*	2.25	0.0006	50	*BIRC6*	2.0	0.0068
25	*FAM101A*	2.23	0.0546	51	*PUS7L*	2.0	0.001
26	*SMG1*	2.22	0.0079				

^a^Unadjusted P < 0.05 for all genes in the table.

**Table 3:** Genes with higher expression levels in Europeans.

	Gene symbol	Fold change	FDR P value^a^		Gene symbol	Fold change	FDR P value
1	*MEGF10*	5.26	0.0685	15	*SSPN*	2.3	0.0159
2	*VIT*	4.02	0.0581	16	*DPYSL4*	2.27	0.0269
3	*SEMA6D*	3.2	0.0459	17	*WISP3*	2.27	0.0115
4	*GIMAP2*	2.95	0.0813	18	*EGR3*	2.25	0.0389
5	*MEOX2*	2.94	0.0217	19	*CCDC102B*	2.24	0.0742
6	*PDXDC1*	2.9	0.039	20	*EGR2*	2.24	0.0122
7	*SAMD5*	2.85	0.0515	21	*FAM43A*	2.19	0.0546
8	*ALDH1A3*	2.71	0.023	22	*TPD52L1*	2.15	0.0202
9	*DAPK1*	2.69	0.0689	23	*BEX4*	2.13	0.0005
10	*MKX*	2.62	0.0171	24	*NOV*	2.12	0.0476
11	*EFHD1*	2.59	0.0079	25	*WFDC1*	2.12	0.0015
12	*GLDN*	2.48	0.0717	26	*RCAN2*	2.09	0.0477
13	*LYPD6B*	2.46	0.0926	27	*BRINP1*	2.07	0.0949
14	*DDO*	2.33	0.0498	28	*LYPD6*	2.03	0.0522

^a^Unadjusted P < 0.05 for all genes in the table.

Further analysis of the microarray results aimed to identify biological processes that are overrepresented in the differentially expressed genes. For this analysis we used a less stringent threshold of unadjusted P < 0.05 instead of the FDR-adjusted P value used above, aiming to include genes that had less stringent differential expression but could be indicative of biological processes of interest when considered as a group. This threshold produced an enriched list of 127 differentially expressed genes (indicated in colour on the scatter plot in Figure S3). Using the PANTHER classification system^12^, we identified 16 biological processes that were overrepresented in this list (FDR-adjusted P value < 0.05 and fold enrichment > 2.5) (Table 4). Biological processes of potential relevance to bone cell biology included fat cell differentiation (ninefold enrichment, with 5 genes represented on the list of differentially expressed genes), positive regulation of cell adhesion (fourfold enrichment, 11 genes), and regulation of cell migration or motility (threefold enrichment, 17 genes).

**Table 4:** Biological processes overrepresented in the differentially expressed genes.

	GO biological process^a^	Fold enrichment	FDR P value
1	Endoderm formation (GO:0001706)	16.1	0.0188
2	Fat cell differentiation (GO:0045444)	9.04	0.0380
3	Angiogenesis (GO:0001525)	5.59	0.0074
4	Blood vessel morphogenesis (GO:0048514)	5.13	0.0033
5	Blood vessel development (GO:0001568)	4.23	0.0141
6	Positive regulation of cell adhesion (GO:0045785)	4.18	0.0380
7	Vasculature development (GO:0001944)	4.04	0.0191
8	Tube morphogenesis (GO:0035239)	3.44	0.0376
9	Anatomical structure formation involved in morphogenesis (GO:0048646)	3.29	0.0111
10	Regulation of cell migration (GO:0030334)	3.14	0.0242
11	Regulation of cell motility (GO:2000145)	2.93	0.0394
12	Regulation of cellular component movement (GO:0051270)	2.84	0.0386
13	Negative regulation of developmental process (GO:0051093)	2.84	0.0491
14	Regulation of anatomical structure morphogenesis (GO:0022603)	2.81	0.0325
15	Epithelium development (GO:0060429)	2.77	0.0386
16	Tissue development (GO:0009888)	2.67	0.0016

^a^GO, Gene Ontology; processes with FDR < 0.05, fold enrichment > 2.5 are presented.

### Expression analysis of selected genes by real-time PCR

The expression levels of four pre-selected osteoblast marker genes and eight additional genes were determined by real-time PCR in 26 RNA samples from the Polynesian group and 23 samples from the European group. The names of these genes and their differential expression, as determined by the microarray analysis, are presented in Table 5.

**Table 5:** Results of microarray analysis of the 12 genes that were selected for further investigation by real-time PCR.

	Gene symbol		Fold change	P value
1	*COL1A1*	Collagen type I alpha 1 chain	1.51	0.00007
2	*ALPL*	Alkaline phosphatase	1.5	0.01
3	*BGLAP*	Bone gamma-carboxyglutamate protein (osteocalcin)	1.08	0.57
4	*IBSP*	Integrin binding sialoprotein	− 1.79	0.27
5	*KRT34*	Keratin 34	2.61	0.0002
6	*CCND1*	Cyclin D1	2.12	0.0017
7	*EFNB2*	Ephrin B2	− 1.66	0.0147
8	*CDH13*	Cadherin 13	− 1.69	0.0203
9	*WISP2*	Wnt1-inducible signaling pathway protein-2	− 1.89	0.0006
10	*NOV*	Nephroblastoma overexpressed	− 2.12	0.0012
11	*DKK1*	Dickkopf 1	− 2.16	0.0078
12	*EFHD1*	EF-hand domain family member D1	− 2.59	0.00004

Data expressed as Log_2_ mean fold change, Polynesian/European.

Rows 1–4, osteoblast marker genes; rows 5–12, other genes.

#### Osteoblast marker genes

**Figure 2:**
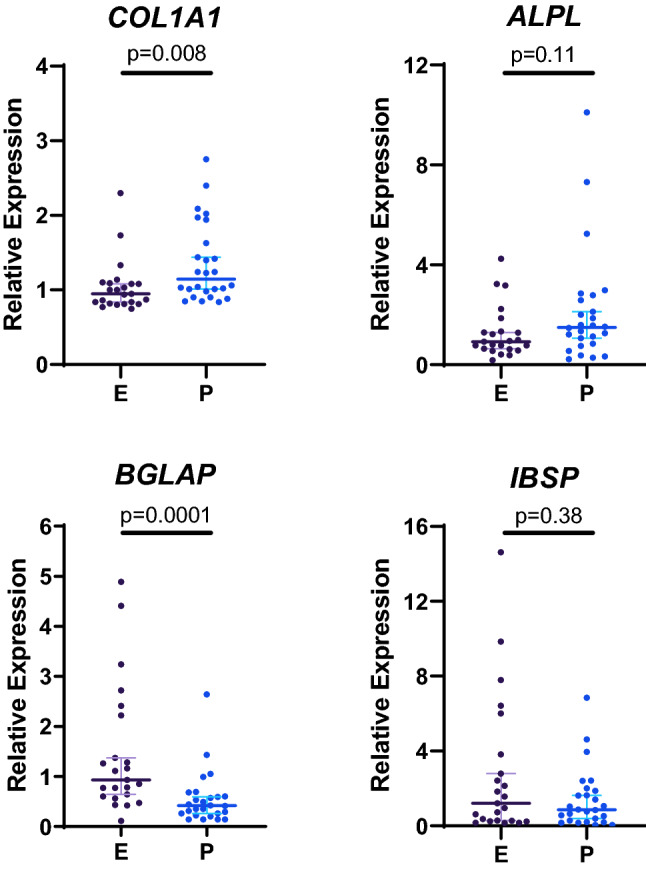
Comparison of relative expression of osteoblast marker genes between the two groups. Gene expression was determined by real-time PCR using TaqMan assays. The graphs present individual values, medians, and 95% CI. Groups were compared by Mann Whitney test. E, European; P, Polynesian. Figure created in GraphPad Prism 8.2.1, https://www.graphpad.com/.

The expression of four osteoblast marker genes are presented in Fig. 2. *COL1A1* (collagen type I, α1 chain) and *ALPL* (alkaline phosphatase) are expressed in osteoblasts from early stages of differentiation*,* and *BGLAP* (osteocalcin), and *IBSP* (integrin-binding sialoprotein) are markers of advanced differentiation^13^. *COL1A1* had higher expression in the Polynesian group, *BGLAP* expression was higher in the European group, and *ALPL* and *IBSP* had similar expression in the two groups. In post hoc analysis with adjustment for age (ANCOVA), *ALPL* expression was significantly higher in the Polynesian group (P = 0.037), and all other comparisons remained unchanged.

#### Other candidate genes

**Figure 3:**
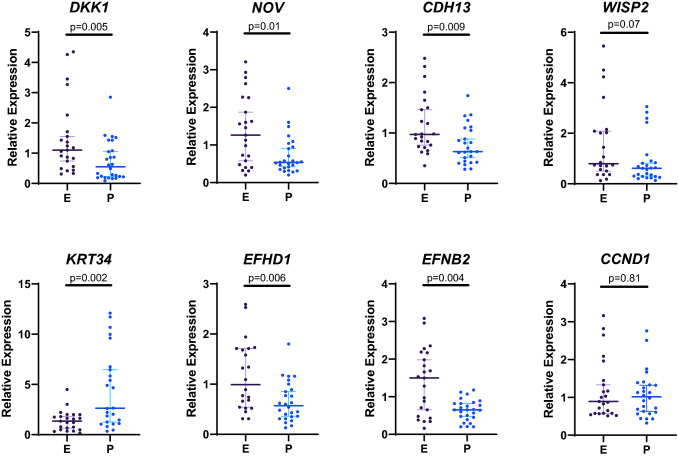
Comparison of relative expression of selected genes between the two groups. Gene expression was determined by real-time PCR using TaqMan assays. The graphs present individual values, medians, and 95%CI. Groups were compared by Mann Whitney test. E, European; P, Polynesian. Figure created in GraphPad Prism 8.2.1, https://www.graphpad.com/.

The selection of the eight additional genes was based on their differential expression, as identified by the microarray analysis, and/or their known roles in bone biology. The proteins encoded by *DKK1*, *CDH13, NOV, CCND1,* and *WISP2* are associated with the canonical Wnt signalling pathway, which plays a central role in the regulation of bone homeostasis. *DKK1*, *CDH13*, and *NOV,* encoding proteins with inhibitory effects on Wnt signalling^14–16^, had higher expression levels in the European group (Fig. 3). *CCND1* and *WISP2* are target genes of the Wnt pathway^17,18^, and their expression levels were not significantly different between the groups.

The remaining three genes tested by real-time PCR were *EFNB2*, *KRT34*, and *EFHD1*. *EFNB2*, encoding EphrinB2, had higher expression in the European group. EphrinB2 is the main ligand of the Eph4 receptor, which is also expressed in osteoblasts. Interactions between ephrinB2/EphB4 in osteoblasts are required for late stage osteoblast differentiation^19^. We also determined the expression of *KRT34* and *EFHD1*, two genes that had no documented activity in bone, and were selected for further analysis because of their differential expression on the microarray. Real-time PCR analysis confirmed the differential expression of these genes, with *KRT34* showing higher expression in the Polynesian group and *EFHD1* showing higher expression in the European group. *KRT34* encodes keratin 34, a type I hair keratin. The scientific literature does not provide much information about the role of KRT34 outside hair^20^. EFHD1 is a calcium-binding protein, localized to the inner mitochondrial membrane^21^. EFHD1 was shown to modulate apoptosis and differentiation of neuronal and muscle precursor cells^22^. Although EFHD1 was not studied in osteoblasts, the expression of the related protein EFHD2 was shown to be upregulated during osteoblast differentiation in human mesenchymal cells^23^.

The results of the differential expression of all eight genes remained similar in post hoc analysis with covariate adjustment for age.

### Expression of *KRT34* and *EFHD1* in differentiating osteoblasts

**Figure 4:**
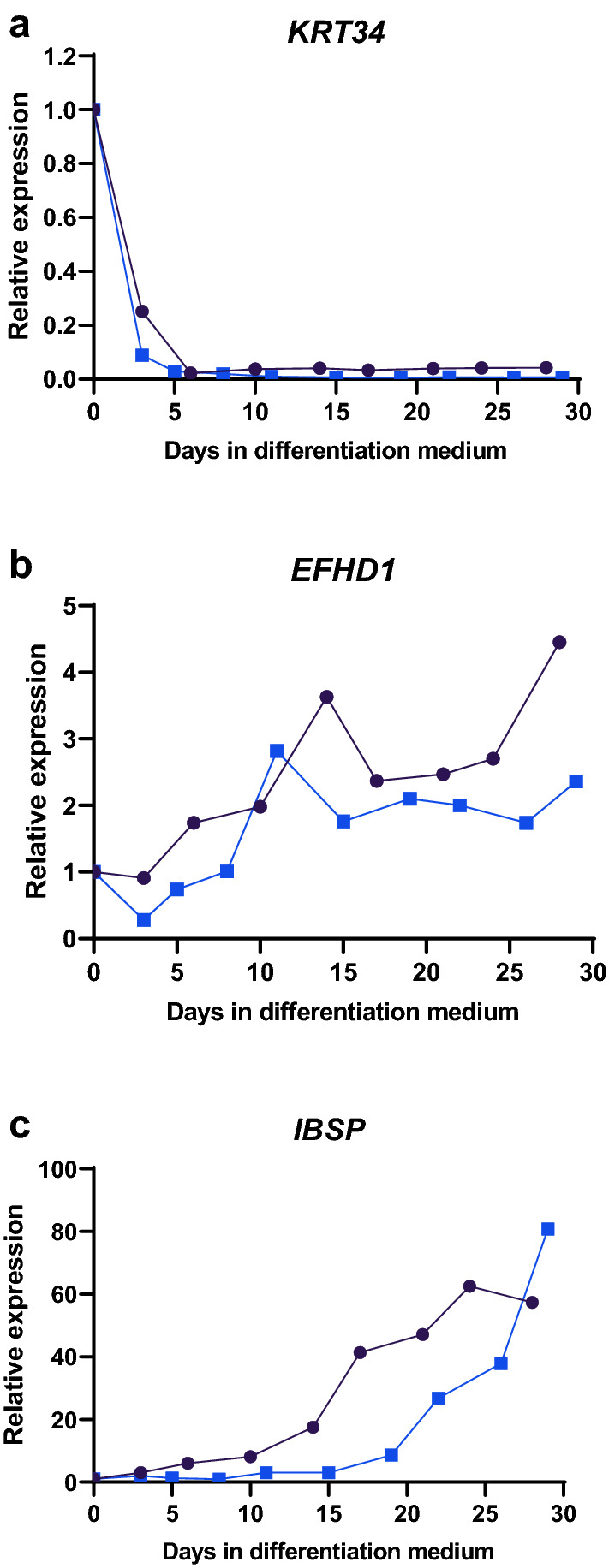
Expression of *KRT34*, *EFHD1, and IBSP* in differentiating osteoblasts. Osteoblasts were cultured for up to 29 days in differentiation medium, and samples were harvested at the indicated time points. Gene expression was determined by real-time PCR. Black line, the level of expression in cells from donor 1; blue line, the level of expression in cells from donor 2. Figure created in GraphPad Prism 8.2.1, https://www.graphpad.com/.

As a preliminary investigation of the expression of *KRT34* and *EFHD1* in osteoblasts, we cultured osteoblasts from two additional donors for up to 29 days under conditions that induce differentiation. The expression of *KRT34* and *EFHD1* was determined at different time points during the differentiation process. *KRT34* was highly expressed in undifferentiated osteoblasts, and its expression dropped sharply and stayed low from day 5 onwards (Fig. 4a), whereas *EFHD1* expression increased through the differentiation period (Fig. 4b). *IBSP* expression was also determined in the cells as an indicator of osteoblast differentiation. *IBSP* expression increased during the culture period, as expected (Fig. 4c).

## Discussion

This study characterised osteoblasts cultured from bone samples that were obtained from patients undergoing joint replacement surgeries for osteoarthritis, and compared between cells that originated from people who identified as Polynesians and those who identified as Europeans. We found no evidence for difference in cell proliferation rate between the groups. Gene expression analysis by microarray identified 79 differentially expressed genes. Further analysis by real time PCR found that *COL1A1* and *KRT34* had higher expression in the Polynesian group, while *BGLAP*, *DKK1*, *NOV*, *CDH13*, *EFHD1*, and *EFNB2* had higher expression in the European group. This differential gene expression may contribute to differences in bone properties that had been determined in these groups in previous studies.

We found that the fraction of cells in S-phase was similar in the two groups, suggesting that the higher BMD in people of Polynesian ancestry does not involve an intrinsic cellular mechanism that accelerates osteoblast proliferation. However, we cannot exclude the possibility that in vivo, differences exist in the proliferative response to extrinsic signals. These potential differences would be missed in our in vitro cultures.

The osteoblast cultures used in this study are primary cultures, and as such are heterogeneous. The method we used for outgrowth cultures from bone fragments is based on the well-established method of growing primary human osteoblastic cells^24^. In previous studies in our lab, we demonstrated alkaline phosphatase activity in the majority of cells^25^. In the current study, the high expression of *BGLAP*, the gene encoding osteocalcin and the most specific osteoblast marker characterised so far, indicated the presence of cells of the osteoblast lineage. The gene expression results suggest that osteoblasts obtained from the two groups vary in their differentiation state under the culture conditions used here. *BGLAP* and *EFNB2*, two genes that are upregulated in later stages of osteoblast differentiation^13,19^, had higher expression in the European group, whereas *COL1A1* had higher expression in the Polynesian group. In addition, *ALPL,* which together with *COL1A1* is highly expressed from early differentiation stages, was higher in the Polynesian group after covariate adjustment for age. The expression profile of *KRT34* and *EFHD1* in osteoblasts have not been described previously, but we found that in long term cultures, under conditions that induced osteoblast differentiation, *KRT34* expression declined with time, while *EFHD1* expression increased. The higher level of *KRT34* and the lower level of *EFHD1* in the Polynesian group are consistent with the observation that the osteoblasts from this group demonstrated a less differentiated phenotype than osteoblasts from the European group. It is possible that a prolonged duration of the earlier differentiation stages allows the osteoblasts to produce larger amounts of extracellular matrix. Interestingly, the level of the late marker *IBSP*^13^ was similar in the two groups and no additional late markers were identified by the microarray analysis, suggesting that a more subtle tuning of differentiation pathways may exist.

The increased levels of *DKK1*, *CDH13,* and *NOV* in the European group suggest an attenuation of the Wnt signalling pathway, which would negatively affect bone formation^14^. The proteins encoded by these genes have inhibitory effects on Wnt signalling^14–16^. *DKK1* has been studied extensively, as the regulation of the Wnt signalling pathway in bone is considered to be predominantly controlled by DKK1 and the other key Wnt inhibitor, sclerostin (*SOST*). *SOST* is only expressed in osteocytes and was undetectable in our osteoblast cultures. *CDH13* encodes cadherin 13, that plays a role in cell adhesion and communication, and has been shown to inhibit Wnt signalling by downregulating the expression of the LRP5 receptor^16^. NOV (CCN3), impairs normal osteoblast differentiation through multiple mechanisms and has been shown to reduce β-Catenin levels and the expression of WNT3 target genes^15^. Further analysis that focuses on components of the Wnt signalling pathway and its targets could potentially determine the contribution of this pathway to ethnic variation in bone.

Three genes identified by the microarray analysis as having over threefold difference between the groups, had been previously described in the musculoskeletal system, and may contribute to differences in bone properties. *HAS1*, which had higher expression in the Polynesian group, encodes hyaluronan synthase 1, a key enzyme in the synthesis of the extracellular matrix polysaccharide, hyaluronan. Hyaluronan is synthesised by both osteoblasts and osteoclasts and plays a role in the regulation of bone remodelling, promoting osteoblast activity and suppressing bone resorption^26^. *MEGF10* and *SEMA6D* had higher expression in the European group. MEGF10 has no known activity in bone, but in muscle it regulates proliferation and migration of myoblasts, via interactions with key components of the Notch signalling pathway^9^. Recently, variants of the *MEGF10* were found to be associated with bone mineral density in children^10^. SEMA6D*,* a member of the large family of semaphorins, is a strong inducer of osteoclastogenesis^11^.

Real time PCR is often used to validate differential gene expression identified by microarray analysis. Here, following the microarray analysis of 10 samples from each group, we determined the expression of 12 genes in 26 samples from the Polynesian group and 23 samples from the European group. In general, the two methods produced congruent results. This excludes *BGLAP*, that had similar expression levels in the two groups in the microarray analysis, and was significantly higher in the European group in the real-time PCR analysis (P < 0.0001), and *WISP2* and *CCND1* that were differentially expressed by microarray analysis and were not significantly different between the groups by real-time PCR.

All the molecular investigations in our study focused on RNA, while genomic DNA was not extracted or used in any of our experiments. Studies of genetic variations among human populations have predominantly focused on DNA polymorphisms, providing valuable data, but leaving the functional significance of the variations mostly unexplored. Gene expression profile is closely associated with the distinctive characteristics of a cell, and therefore with its function. Several large-scale gene expression studies that determined differences among human populations used information and samples from the International HapMap Project^27–29^. The relation between genome variation and gene expression suggested that gene expression is heritable, and varies in different human populations. Other studies used a similar approach to that used in our study. For example, a microarray analysis of gene expression in adipose and skeletal muscle tissue samples obtained from European Americans and African Americans, identified 58 differentially expressed genes in muscle and 140 genes in adipose tissue^30^. To the best of our knowledge, gene expression in bone samples obtained from different human populations has not been examined previously.

By comparing osteoblast properties between Polynesians and Europeans, we aimed to identify molecular mechanisms that contribute to a better bone quality and the lower risk of fracture in the Polynesian population^7^. Fracture risk is an outcome of multiple factors. Two of these factors, body composition and BMD, have been previously shown to differ between Polynesians and Europeans. People of Polynesian ancestry in Aotearoa New Zealand have a high prevalence of obesity, with greater lean mass and fat mass in comparison to Europeans^31–33^. However, even when groups were weight-matched, Polynesian women had higher BMD at all skeletal sites in comparison to European women^5,6^. Given the positive relations between weight and BMD, these findings suggest that the overall higher BMD in Polynesian people results from a combination of weight-dependent and weight-independent mechanisms. As the two groups in our study had similar BMI, our result are mostly relevant to potential mechanisms that are independent of weight.

Our study had several limitations. The grouping of the participants by ancestry/ethnicity must be considered carefully. Although ethnic differences in bone density and fracture risk are well documented both globally and in Aotearoa New Zealand, there is little evidence that ethnic categories relate to genetic identity^34^. In fact, genetic studies consistently find more genetic diversity within ethnic groups than between them^34,35^. We acknowledge that the ‘self-identified ancestry/ ethnicity’ used in our study has no accurate biomedical meaning and refers predominantly to a social construct^34^. Another potential limitation of our study is the age difference between the groups analysed for cell fraction in S-phase and gene expression by real time PCR. This limitation was addressed by post hoc sensitivity analysis with covariate adjustment for age, as presented in the “Results” section. Finally, obtaining the bone samples from patients undergoing joint replacement surgeries for OA can be considered as a limitation of the study. Pathological mechanisms in OA affect the whole joint, and characteristic structural changes in bone can be seen in the subchondral region^36^. Although we avoided subchondral regions when excising the bone samples, we cannot exclude the possibility that the samples were affected by OA. However, OA was not considered a confounding factor in our study, as all the participants had severe OA that required joint replacement surgery.

In summary, our study identified differentially expressed genes in osteoblasts cultured from bone samples obtained from Polynesian and European people. The genes identified here suggest that the duration of stages along the osteoblast differentiation pathway vary between the two groups, and the regulation of the Wnt signalling pathway is altered. These results suggest that there are intrinsic differences between the osteoblast-lineage cell populations obtained from the two groups in this study, but further studies are required to link changes in gene expression, genetics and bone properties.

## Methods

### Participants

Ethical approval for the study was obtained from the New Zealand Northern A Health and Disability Ethics Committee. All experiments were performed in accordance with the guidelines and regulations of the Committee and all participants provided written informed consent. Participants were undergoing total hip or knee replacement surgeries for osteoarthritis. Exclusion criteria included the use of bone-active medications, metabolic bone disease and fracture of the hip to be replaced. Group allocation was by self-identified ethnicity. The European group included people of European ancestry living in Aotearoa New Zealand. The group of people of Polynesian ancestry living in Aotearoa New Zealand included individuals who identified as Samoan (n = 15), NZ Māori (n = 10), Niuean (n = 2), Cook Island Māori (n = 2) and Tongan (n = 1).

### Osteoblast outgrowth cultures

Trabecular bone removed during surgery was cut into small fragments, rinsed several times with PBS and digested with 1 mg/mL collagenase (C6885, Sigma-Aldrich, MO, USA) for 30 min at 37 °C. Bone fragments were then incubated in DMEM/10% FBS (Thermo Fisher Scientific, MA, USA) and 5 μg/mL l-ascorbic acid 2-phosphate (A2P) (Sigma-Aldrich) at 37 °C, 5% CO_2_ until outgrowth of cells was noted, usually 3–4 days later. Bone fragments were transferred to new flasks and cultured with media replacement every 3–4 days. When the cells were approximately 50% confluent they were harvested for cell cycle analysis and the bone fragments were transferred to new flasks, where the outgrowth of osteoblastic cells continued under the same culture conditions until the cells were 90–100% confluent. Seventy-two hours before the end of the culture period, all bone pieces were removed from the flasks and media renewed.

### Analysis of cell cycle by flow cytometry

After harvesting the cells by trypsin digestion, cell pellets were washed once in PBS and then in PBS/1%FCS and resuspended in 200µL of ice cold PBS/1%FBS. Cells were fixed by dropwise addition of 2 mL of ice cold 100% methanol and stored at − 20 °C. Fixed cells were rehydrated by three washes in cold PBS/3%FBS and resuspended in 1 mL PBS/3%FBS. Before analysis, cells were incubated for 10 min with 100 µg/mL RNase and 20 µg/mL propidium iodide (Thermo Fisher Scientific). Analysis of cell DNA content was performed using a BD (Mountain View, CA, USA) FACScan flow cytometer with 10,000 events collected for each analysis. Cell cycle distribution was calculated with the ModFit LT software (Verity Software House, Topsham, ME, USA). Cell cycle was analysed in 21 osteoblast cultures from the Polynesian group and 22 from the European group.

### Gene expression profiling

Genomic DNA has not been extracted or investigated in this study. RNA was extracted from cells using the RNeasy Mini-prep Kit with in-column DNase digestion (QIAGEN). RNA quality was determined with RNA StdSens Kit on Experion Automated Electrophoresis System (Bio-Rad). Ten RNA samples from each group, with RNA quality indicator values of 9.7–10, were selected for microarray analysis. Biotinylated, fragmented cRNA was prepared from 210 ng of total RNA using the GeneChip 3′ IVT Express Kit (Affymetrix). cRNA samples were hybridized to GeneChip PrimeView Human Gene Expression Arrays (Affymetrix). Microarray hybridisation, scanning and quality control were carried out at the Auckland Genomics Centre, Faculty of Sciences, University of Auckland.

### Real time PCR

Four of the bone samples collected from Polynesian participants and two samples from European participants were excluded from the gene expression analysis because of low RNA quality indicator values, leaving 26 samples from the Polynesian group and 23 from the European group. cDNA was synthesised with SuperScript III Reverse Transcriptase (Thermo Fisher Scientific) from 500 ng of RNA. Gene expression was analysed in multiplex PCR with VIC-labelled TaqMan assay for 18S rRNA as the endogenous control and FAM-labelled TaqMan assay for the target genes (Thermo Fisher Scientific), using the 2^−ΔΔCt^ method.

### Osteoblast differentiation

Human osteoblasts grown from bone fragments were seeded in αMEM/10% FBS and 50 µg/mL A2P at a density of 1.5 × 10^5^ cells/well in 6-well plates (Day 0). Once cells reached confluence, about 3 days later, media were changed to αMEM/5% FBS, 50 µg/mL A2P, 10 mM β-glycerophosphate, and 10 nM dexamethasone. Media were changed twice weekly, and cells were maintained for up to 4 weeks. Cells were harvested for RNA extraction at the indicated time points. From day 10 onwards, cells were treated with 120 µg/mL collagenase for 30 min at 37 °C before cell harvest.

### Statistical analysis

Transcriptome Analysis Console 4.0.2 (Affymetrix, Inc., Thermo Fisher Scientific, CA, USA) was used for microarray analysis. Differential gene expression was analysed by the Limma Bioconductor package (implemented in Transcriptome Analysis Console 4.0.2), using the Robust Multi-chip Analysis (RMA) algorithm for background adjustment, quantile normalisation, and summarisation. False discovery rate (FDR)-adjusted P values were calculated to control for multiple comparisons. No further adjustments for multiple comparisons were performed. The PANTHER classification system^12^ was used for classification and enrichment analyses. GraphPad Prism 8.2.1 (GraphPad Software, La Jolla CA, USA) and SAS software (v9.4, SAS Institute Inc, Cary, NC, USA) were used for all other statistical analyses. The statistical tests used are indicated in the figure legends. Post hoc sensitivity analysis was used for the cell cycle and real-time PCR experiments, with adjustment for age (ANCOVA). There was no significant age difference between the two groups analysed by microarrays. Experiments described under cell cycle by flow cytometry and gene expression by real-time PCR were repeated at least twice, and the means of these repeats are presented for each sample in the scatter plots. Tests were 2-tailed with a 5% significance level.

## Supplementary Information

Supplementary Information.

## Data Availability

Microarray data were submitted to the GEO repository. The records have been assigned the GEO accession number GSE157322.
