# Supplementary material for: Molecular characterisation of osteoblasts from bone obtained from people of Polynesian and European ancestry undergoing joint replacement surgery
Source: Sci Rep. 2021 Jan 28;11:2428. doi: 10.1038/s41598-021-81731-5 (PMC7844412; doi:10.1038/s41598-021-81731-5)
Supplement: Supplementary file 1 — Supplementary Information. [file 41598_2021_81731_MOESM1_ESM.pdf]

Molecular characterisation of osteoblasts from bone obtained from people of Polynesian and European ancestry undergoing joint replacement surgery

Naot D, Bentley J, Macpherson C, Pitto RP, Bava U, Choi AJ, Matthews BG, Callon CE, Gao R, Horne A, Gamble GD, Reid IR, Cornish J

Figure S1

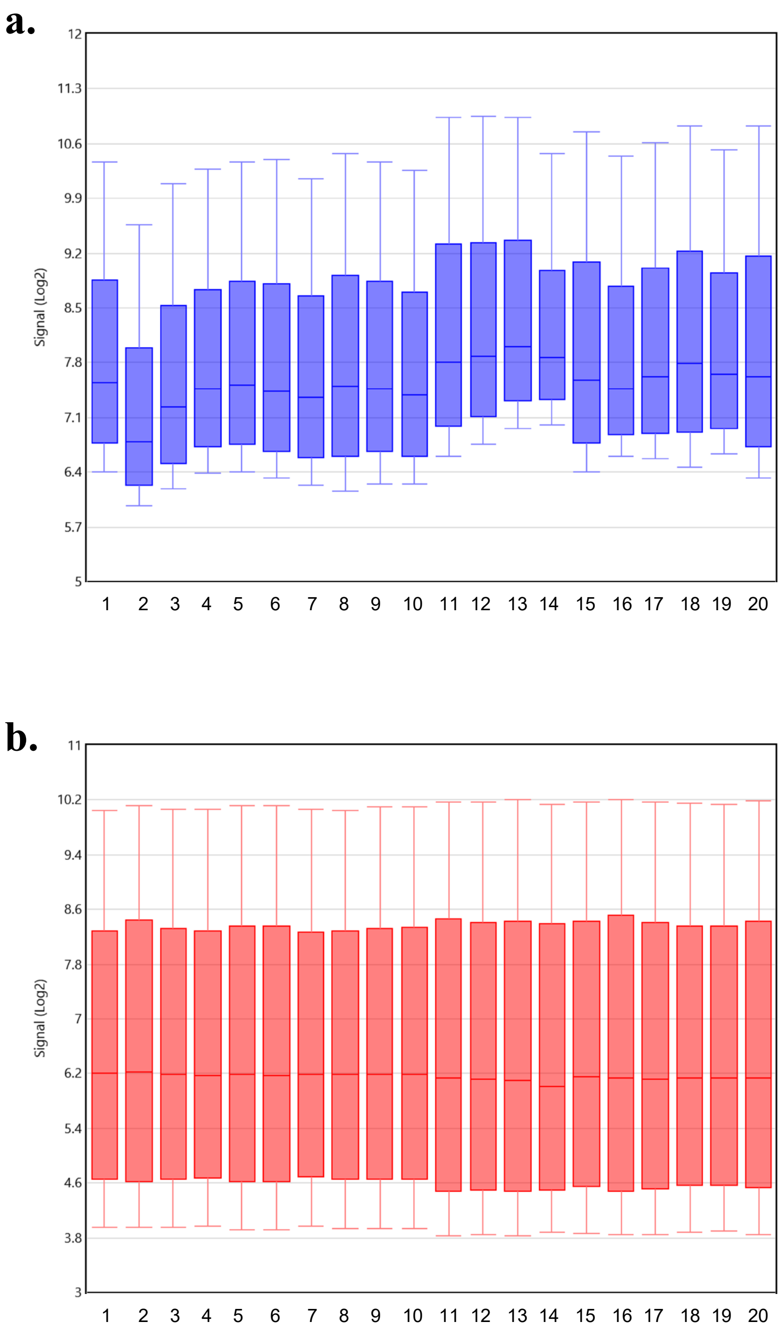

Figure S1 Microarray signal box plot (a) Raw data (CEL files) (b) Normalised data (CHP files). Each box plot indicates the signal distribution in one array; whiskers represent 10th to 90th percentile. Arrays 1-5 and 11-15, samples from Polynesian participants; arrays 6-10 and 16-20, samples from European participants.
Figure was created in Transcriptome Analysis Console 4.0.2
https://www.thermofisher.com/nz/en/home/life-science/microarray-analysis/microarray-analysis-instruments-software-services/microarray-analysis-software.html

Figure S2

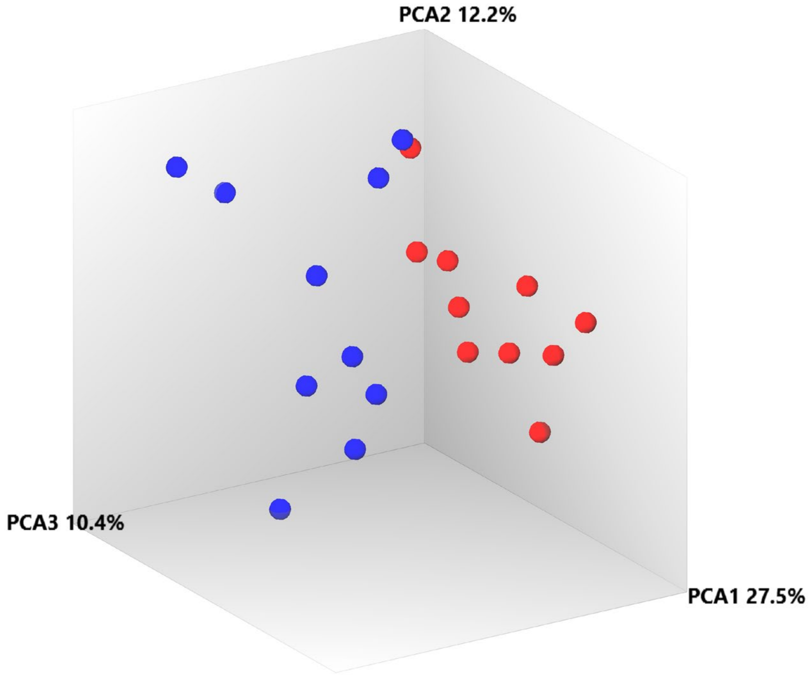

Figure S2 Principal component analysis of gene expression data Principal components 1, 2 and 3 accounted for 50.1% of the variation between the two groups.
Blue, samples from Polynesian participants; Red, samples from European participants.
Figure was created in Transcriptome Analysis Console 4.0.2
https://www.thermofisher.com/nz/en/home/life-science/microarray-analysis/microarray-analysis-instruments-software-services/microarray-analysis-software.html

Figure S3

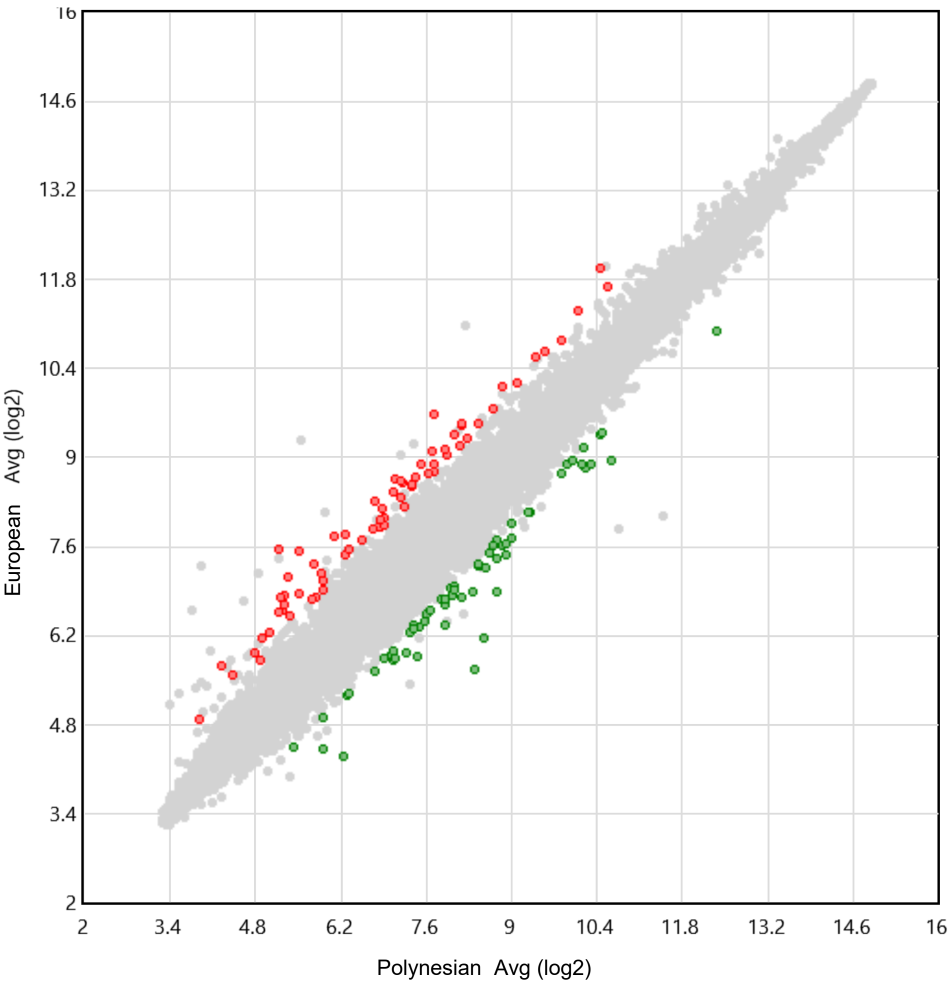

Figure S3 Signal scatter plot Differential gene expression analysis using fold change >2 and unadjusted P <0.05 as a threshold. Genes with higher expression in the European group are labelled in red, gene with higher expression in the Polynesian group are in green.

Figure was created in Transcriptome Analysis Console 4.0.2 https://www.thermofisher.com/nz/en/home/life-science/microarray-analysis/microarray-analysis-instruments-software-services/microarray-analysis-software.html
